# Brain Microbiota in Huntington’s Disease Patients

**DOI:** 10.3389/fmicb.2019.02622

**Published:** 2019-11-12

**Authors:** Ruth Alonso, Diana Pisa, Luis Carrasco

**Affiliations:** Centro de Biología Molecular “Severo Ochoa” (CSIC-UAM), Universidad Autónoma de Madrid, Madrid, Spain

**Keywords:** Huntington’s disease, neurodegenerative diseases, polymicrobial infections, endomycosomes, fungal infection, next generation sequencing

## Abstract

One of the most important challenges facing medical science is to better understand the cause of neuronal pathology in neurodegenerative diseases. Such is the case for Huntington’s disease (HD), a genetic disorder primarily caused by a triplet expansion in the *Huntingtin* gene (*HTT*). Although aberrant *HTT* is expressed from embryogenesis, it remains puzzling as to why the onset of disease symptoms manifest only after several decades of life. In the present study, we investigated the possibility of microbial infection in brain tissue from patients with HD, reasoning that perhaps mutated *HTT* could be deleterious for immune cells and neural tissue, and could facilitate microbial colonization. Using immunohistochemistry approaches, we observed a variety of fungal structures in the striatum and frontal cortex of seven HD patients. Some of these fungi were found in close proximity to the nucleus, or even as intranuclear inclusions. Identification of the fungal species was accomplished by next-generation sequencing (NGS). Interestingly, some genera, such as *Ramularia*, appeared unique to HD patients, and have not been previously described in other neurodegenerative diseases. Several bacterial species were also identified both by PCR and NGS. Notably, a curved and filamentous structure that immunoreacts with anti-bacterial antibodies was characteristic of HD brains and has not been previously observed in brain tissue from neurodegenerative patients. Prevalent bacterial genera included *Pseudomonas*, *Acinetobacter*, and *Burkholderia*. Collectively, our results represent the first attempt to identify the brain microbiota in HD. Our observations suggest that microbial colonization may be a risk factor for HD and might explain why the onset of the disease appears after several decades of life. Importantly, they may open a new field of investigation and could help in the design of new therapeutic strategies for this devastating disorder.

## Introduction

Huntington’s disease (HD) is a heritable neurodegenerative disease with autosomal dominance (Caron et al., 2018; Testa and Jankovic, 2019) that is characterized by degeneration of neurons in specific brain regions. Its incidence fluctuates between different populations, being higher in people of Western European descent, with the exception of some ethnic groups in Iberoamerica. HD has an estimated prevalence of 5–12 per 100,000 persons in Europe and 0.1–2 per 100,000 persons of Asian and African descent (Paradisi et al., 2008; Pringsheim et al., 2012; Apolinario et al., 2017; Paz et al., 2017). Disease symptoms include motor alterations, cognitive decline and psychiatric disorders that can progress to dementia (Jimenez-Sanchez et al., 2017). Choreic movements are typical in the majority of patients, together with gait disturbances, rigidity, behavioral changes and depression (Caron et al., 2018). The disease is usually diagnosed when motor problems are apparent, typically between 35 and 55 years of age, although prodromal symptoms may occur 10–15 years before diagnosis (Dayalu and Albin, 2015). Degeneration of neurons in the caudate and putamen and in the cerebral cortex is the primary pathologic hallmark of HD (Waldvogel et al., 2015). In addition, widespread atrophy is common, leading to a significant reduction in the brain volume, and affecting several regions of the central nervous system (CNS), including the subthalamic nucleus, thalamus, hypothalamus, globus pallidus, hippocampus, and substantia nigra (Caron et al., 2018).

A breakthrough in HD research came in 1983 with the discovery of a genetic marker of the disease near the tip of the short arm of chromosome 4 (Gusella et al., 1983), and 10 years later a triplet expansion repeat in the novel gene *Huntingtin* (*HTT*) was mapped as the causal mutation for HD (MacDonald et al., 1993; Holmans et al., 2017). Expansion of the CAG triplet (encoding for glutamine) can be variable (greater than 40 times in the mutated gene), which leads to the synthesis of a long polyglutamine (polyQ) tract at the amino terminus of the protein Huntingtin. In normal subjects the CAG repeat length can vary between 7 and 26, and CAG repeats in the range 27–35 usually does not confer the disease phenotype, but these individuals are prone to CAG repeat instability and the consequent probability of having a child with an allele in the pathogenic CAG range (Semaka et al., 2013; Kay et al., 2018). An expansion in one allele of 36–39 repeats confers risk for HD, but symptoms may not develop. In fact, elderly subjects with CAG repeats in this range without HD symptoms are common (Kay et al., 2016). CAG repeat expansion beyond 40 results in full penetrance of the disease, which will manifest during lifetime (Holmans et al., 2017). In this regard, very long triplet repeats (usually >60) can lead to a juvenile onset of HD. Accordingly, there is an inverse correlation between the length of the triplet repeats and the age at which the first symptoms of HD appear. Thus far, there is no therapy to prevent or to modify the progression of HD and pharmacological treatment is aimed at managing the symptoms of the disease (Dickey and La Spada, 2018). Nevertheless, a number of new strategies are being explored to block the synthesis of the mutated Huntingtin, for example, using small interfering RNAs or antisense oligonucleotides (Kim and Kim, 2014; Singh et al., 2016; Lane et al., 2018).

Huntingtin is a large protein of 359 kDa that is subject to a number of post-translational modifications, including phosphorylation, acetylation, palmitoylation, among others (Saudou and Humbert, 2016), and contains several domains that can interact with other macromolecules. In this respect, Huntingtin is considered a scaffolding protein that may act as a platform for other proteins, and is capable of modulating a variety of cellular functions, including transcription, vesicular trafficking, autophagy, cell division, among other functions (Saudou and Humbert, 2016). Huntingtin is necessary for early development as the knock-out for *HTT* is embryonic lethal in mice (White et al., 1997; Reiner et al., 2001). Also, *HTT* is ubiquitously expressed in many tissues, and the presence of the mutated protein could affect normal physiological functions, particularly in CNS tissues and the immune system. It is believed that mutated Huntingtin is more prone to proteolytic cleavage, and can generate an amino fragment with toxic properties (Ratovitski et al., 2009; Tebbenkamp et al., 2012), but it remains controversial whether the formation of mutated Huntingtin aggregates or its moieties is detrimental or beneficial for cell survival (Saudou and Humbert, 2016). It is plausible that these aggregates sequester the deleterious soluble Huntingtin bearing the long polyQ tract, and thus improves cellular functions and survival. Nevertheless, the accepted hypothesis in HD pathology is that the synthesis of mutated Huntingtin may lead to cell death, and thus to the destruction of neurons, particularly in the striatum and the cortex, although other regions in the CNS and other tissues can be affected. It remains puzzling as to why the pathological symptoms of HD manifest after decades of *HTT* expression, and also why the CNS is especially vulnerable while other tissues that express *HTT* may be less affected.

An emerging field of research in neurodegenerative diseases is the link between the gut microbiota and the CNS (Forbes et al., 2018; Ma et al., 2019; Roy Sarkar and Banerjee, 2019). Although some microbial infections have been studied as potential risk factors in a variety of neurodegenerative diseases (Alonso et al., 2015, 2017b, 2018b; Pisa et al., 2015b, 2017; Carrasco et al., 2017), to our knowledge, no studies have analyzed the potential involvement of microorganisms as modulators of the severity of HD. Several arguments support such an analysis in HD: (1) HD is associated with neuroinflammation, with the consequent increase in cytokine levels and microglia activation (Crotti and Glass, 2015; Rocha et al., 2016), which also occurs after infection; (2) synthesis of amyloid is augmented in HD, as occurs in other neurodegenerative diseases such as Alzheimer’s disease and amyotrophic lateral sclerosis (Haass and Selkoe, 2007; Shao et al., 2017). Amyloid peptide has anti-microbial activity, stimulates the innate immune system, and its synthesis is believed to be triggered by microbial infections (Soscia et al., 2010; Kumar et al., 2016); (3) *corpora amylacea* (CA) can be found in HD brains, as occurs with other neurodegenerative disorders (Pisa et al., 2016a). We recently advanced the idea that CA could also be a response to microbial infections, as these bodies can collect/scavenge microbial debris (Pisa et al., 2018); (4) the fungi and bacteria present in the CNS of patients with several neurodegenerative diseases, but not in HD patients, have been identified and analyzed in detail in the last few years (Pisa et al., 2015a, 2017; Alonso et al., 2017a, 2018a).

Based on this information, we considered that it would be interesting to compare the brain microbiota of these diseases with that of patients with HD. In principle, it could be possible that the modifications in cell functioning by the expression of mutated Huntingtin could influence the microbial colonization of the CNS. Thus, the present study aimed to comprehensively test for potential fungi and bacteria in the CNS of HD patients.

## Materials and Methods

### Brain Samples From HD Patients

Brain paraffin sections and frozen tissue were obtained from seven patients diagnosed with HD. Samples were obtained from two regions: the striatum and the frontal cortex (FC). The age and gender of each patient are listed in Supplementary Table 1. Based on the grade of lesions found in the striatum in the different HD patients they were classified from grade 0 to grade IV (Supplementary Table 1; Vonsattel et al., 1985). The *Banco de Tejidos* CIEN, Madrid, supplied the samples and they were analyzed anonymously. All brain samples were provided by the same laboratory. All of them were processed according to a common postmortem protocol followed by Banco de Tejidos CIEN. Briefly, rapid neuropathological autopsy was performed upon call by the donor’s proxies (mean postmortem interval, 4.5 h). Immediately after extraction, the right half of the brain was sliced and frozen at –80°C. The left half was fixed by immersion in phosphate-buffered 4% formaldehyde for at least 3 weeks. A full neuropathological study was performed in the left half brain after fixation. Neuropathological diagnosis and staging of all disease entities was performed according to consensus criteria. Samples from the frozen tissue were obtained with sterile instruments taking all measures to avoid contamination in a laminar flow hood. Sample transfer was carried out according to national regulations concerning research on human biological samples and written informed consent was obtained in all cases. The study was approved by the ethic committee of the Universidad Autónoma de Madrid (Ref. number CEI-60-1066). To avoid contamination, the frozen tissue was handled with sterile instruments in a laminar flow hood.

### Antibodies Employed in This Work

The following rabbit polyclonal antibodies used in the present study have been described previously (Pisa et al., 2015a, 2016a,b): against *Candida albicans* (used at 1:100 dilution), *Candida glabrata* (1:500 dilution), *Syncephalastrum racemosum* (1:100 dilution), *Penicillium notatum* (1:100 dilution), *Phoma betae* (1:100 dilution), and against purified fungal enolase (1:50 dilution). A rabbit polyclonal antibody against fungal chitin (1:50 dilution) was generously provided by Dr. M.N. Horst (Mercer University, Macon, GA, United States). The remaining antibodies employed were purchased from commercial vendors: rabbit polyclonal antibody against *Borrelia burgdorferi* (Genetex, Irvine, CA, United States) used at 1:50 dilution; mouse monoclonal antibody against *B. burgdorferi* (Abcam, Cambridge, United Kingdom) used at 1:10 dilution; mouse peptidoglycan monoclonal antibody (Thermo Fisher Scientific, Waltham, MA, United States) used at 1:20 dilution; and a rabbit polyclonal antibody against *Chlamydophila pneumoniae*, which immunoreacts with the major outer porin (Biorbyt, Cambridge, United Kingdom) used at 1:20 dilution.

### Analysis of CNS Sections From HD Patients by Immunohistochemistry

Standard techniques were used for paraffin embedding and sectioning of CNS tissue. Protocols for immunohistochemical analysis have been described (Pisa et al., 2016b). Most of the images were obtained with a Zeiss LSM710 confocal laser scanning microscope equipped with the upright Axio Imager.M2 stand (Zeiss), running ZEN 2010 software, or with a Zeiss LSM800 confocal laser-scanning microscope equipped with an Axio Observer (Zeiss) inverted microscope and running Zen Blue 2.3 software. Images were deconvoluted using Huygens software (4.2.2 p0) and visualized with ImageJ (NIH).

### DNA Extraction From CNS Tissue

DNA was extracted from frozen tissue obtained from striatum and FC regions of seven HD patients, as described previously (Alonso et al., 2017a).

### Nested PCR Assay

To prevent PCR contamination, we used separate rooms and glassware supplies for PCR set-up and products. We also used aliquoted reagents, positive-displacement pipettes, aerosol-resistant tips and several negative controls. To assay for bacterial DNA, we used nested PCR with several primer pairs as described (Alonso et al., 2017a), which amplify a region between the V3 and V4 variable region of the prokaryotic 16S rRNA gene. In the first PCR, 4 μl (5–10 ng) of DNA was denatured at 95°C for 5 min, followed by 35 cycles of 1 min at 94°C, 1 min at 56°C, and 3 min at 72°C, using primers 27F and 1492R. The second PCR was performed using 2 μl of the product obtained in the first PCR with forward V3 and reverse V4 internal primers, for 30 cycles of 1 min at 94°C, 1 min at 55°C, and 3 min at 72°C.

In addition, the intergenic spacer (IGS) region between rRNA genes of the prokaryotic genome was amplified using the primers 1406 (F)/559 (R) and 1492 (F)/242 (R) in the first and second round PCR, respectively. The first PCR was carried out with 4 μl (5–10 ng) of DNA denatured at 95°C for 5 min, followed by 45 cycles of 1 min at 94°C, 1 min at 55°C, and 3 min at 72°C. The second PCR was performed using 2 μl of the product obtained in the first PCR, for 35 cycles of 1 min at 94°C, 1 min at 57°C, and 3 min at 72°C. Some PCR products were sequenced by Macrogen Inc. (Seoul, South Korea). The sequences have been submitted to the European Nucleotide Archive with the access numbers ERZ964645. The *Borrelia* spp. *flagellin* gene was amplified as described previously (Pisa et al., 2017). The CAG triplets in the HTT gene were tested using the primers described by Yoon et al. (2003). We also analyzed the hexanucleotide expansion repeat in the C9Orf72 gene in the different samples, as described previously (Alonso et al., 2019).

### Next-Generation Sequencing

We used a metagenomics next-generation sequencing (NGS) protocol (Illumina platform) to amplify the ITS1 region of fungi and the 16S rRNA gene of bacteria. This analysis was performed by the Genomics Unit at the Scientific Park of Madrid. Quality analyses were performed over reads using FastQC software^1^. All sequences have been submitted to European Genome-phenome Archive with the accession number EGAS00001003678.

For fungal DNA, the region between the internal 1 primer pair was amplified with specific primers joined to linker sequences in a first round of PCR (specific product of ∼300 nt). A second PCR was performed on this product using fusion primers containing Illumina and linker sequences. For bacterial DNA, primers were designed to amplify the region between V3 and V4 of 16S rDNA gene. These primers were joined to linker sequences in a first round of PCR (specific product of ∼400 nt). A second PCR was performed on this product using fusion primers containing Illumina and linker sequences. PCR products were sequenced on a MiSeq sequencing platform (Illumina).

### Computational Analysis

The adapters from the sequences were deleted using Cutadapt and all sequences with a length shorter than 35 bp were discarded. A minimum read length of 35 bases was set due to the wide range of amplicon lengths (from 35 to 301) with an average of 267 and 256 pb in ITS and 16S samples, respectively. The QIIME^2^ software pipeline was used for metagenomics analysis (Caporaso et al., 2010) including the following steps: quality filtering, clustering sequences to define operational taxonomic unit (OTU), obtaining representative sequences and aligning them against the reference database in order to perform the taxonomic assignment, phylogenetic reconstruction, and diversity analyses. To define the OTUs, the QIIME *pick_open_reference_otus.py* workflow was used with an identity percentage of 97 and 94% in fungi and bacteria, respectively. Regarding the fungal analysis according to the taxonomical classification (species level), we found that on average 42% of the matches corresponded to “uncultured fungus blast” hit. For this reason, an additional standard Blast search analysis was performed. All efforts have been made to avoid contamination during the handling of samples. In fact, in the PCR assays, a control without DNA indicated that it was clean without any apparent DNA fragment, suggesting that no environmental contamination occurred. However, since these negative controls were not included in the DNA sequencing runs, the possibility that some OTUs found are due to contamination can not be completely discarded.

### Principal Component Analysis

The β-diversity parameter (community structure), which gives a between-sample diversity comparison, and core diversity analyses, was performed with QIIME together with a Principal Coordinates Analysis (PCoA) with Bray-Curtis distances. The 3D plot model of the PCA was made with the scatter plot 3D package in R.

## Results

### Analysis of the CAG Expansion in *HTT* and the Hexanucleotide Repeat in C9Orf72

Since the underlying genetic defect in HD is the presence of an expanded CAG repeat in *HTT*, we tested for this in the seven patients studied in this work using nested PCR (Yoon et al., 2003). Gel electrophoresis analysis of the PCR products revealed the presence of two major DNA fragments for each patient: one of a lower size corresponding to the normal allele and one of a higher size corresponding to the mutated allele (Supplementary Figure 1, panel A). After sequencing the PCR products, the number of CAG repeats in each patient in both alleles was determined (Supplementary Table 2). The highest expansion corresponded to 40 CAG repeats in patient HD6. The expansion varied from 35 to 39 repeats (HD1, HD3–HD5) in other patients, and was within normal limits (23 repeats) in patient HD2. In patient HD7, we found a repeat of 17 CAG triplets followed by 17 non-CAG codons and then a repeat of 7 additional CAGs (see sequence in Supplementary Table 2). The number of CAG triplets in the normal allele from the seven HD patients ranged from 7 to 25. The finding that some patients classified as HD according to their clinical symptoms did not have an abnormal expansion repeat has been reported previously (Mariani et al., 2016; Bourinaris and Houlden, 2018).

The C9Orf72 hexanucleotide expansion repeat has been associated with frontotemporal degeneration and amyotrophic lateral sclerosis spectrum disorders (van der Meer et al., 2015). It is also indicated as a possible contributor to several neurodegenerative diseases, including HD. Indeed, patients who presented with an HD-like phenotype and tested negative for pathogenic expansions in *HTT* have been analyzed in this respect (Hensman Moss et al., 2014; Koutsis et al., 2015; Bourinaris and Houlden, 2018), with frequencies of the hexanucleotide expansion ranging from 1.75 to 5%. Thus, to complement our results on the expansion repeat in *HTT*, we performed a direct PCR assay to analyze the hexanucleotide expansion in C9Orf72 of the seven HD patients (Alonso et al., 2019). One major DNA fragment for each patient was obtained after PCR amplification (Supplementary Figure 1, panel B), which was extracted from the agarose gel and sequenced. Our results indicated that in all cases, only three hexanucleotide repeats were obtained (Supplementary Table 2). This number is well below the threshold of 30 repeats believed to be pathogenic (Cleary et al., 2016). In conclusion, none of the seven HD patients exhibited a pathogenic expansion repeat in C9Orf72, and two of them (HD2 and HD7) had a CAG repeat in *HTT* within the normal limits, although patient HD7 contained an abnormal sequence in this region.

### Detection of Fungal Structures in Brain Sections From Huntington’s Disease Patients

**FIGURE 1:**
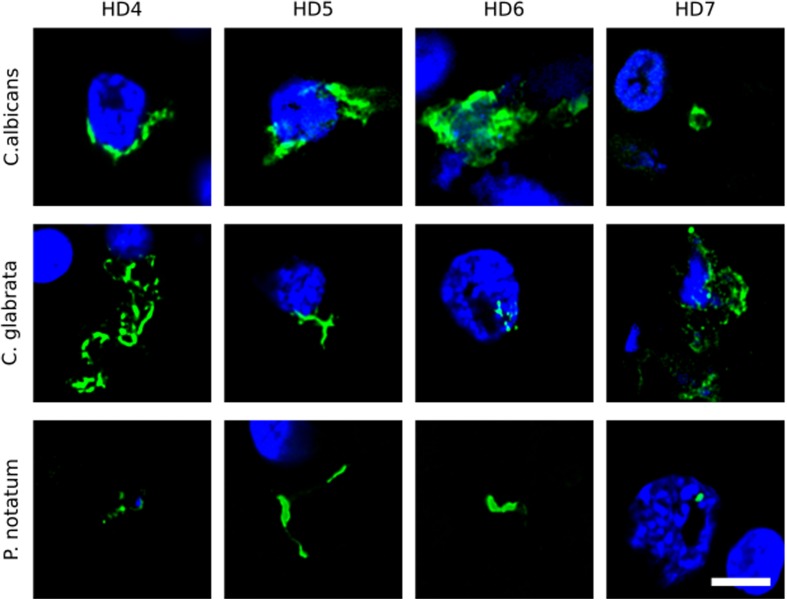
Immunohistochemistry of striatum sections from Huntington’s disease patients using a battery of antifungal antibodies. The striatum CNS region of four HD patients (HD4–HD7) was processed for immunohistochemistry as described in section “Materials and Methods.” Paraffin sections were immunostained with rabbit polyclonal antibodies against *C. albicans, C. glabrata*, and *P. notatum* (green). Nuclei were stained with DAPI (blue). Scale bar: 5 μm.

We first tested for the presence of fungi in HD brains using paraffin sections of striatum tissue from four HD patients (patients HD4–HD7; grades III and IV). To this end, immunofluorescence analysis was performed using three different rabbit polyclonal anti-fungal antibodies, anti-*C. albicans*, anti-*C. glabrata* and anti-*P. notatum* (green), and sections were counterstained with DAPI to mark nuclei (blue). As shown in Figure 1, some hyphal, mycelial and rounded forms were positively stained (green) usually close to the neural cell nucleus (panels HD4 and HD5 of *C. albicans* and *C. glabrata* antibodies), or even apparently in the interior of the nucleus (panels HD6 of *C. albicans* and *C. glabrata*, and HD7 of *C. glabrata* and *P. notatum* antibodies). Of note, these rabbit polyclonal anti-fungal antibodies are not specific for one fungal species and they can cross-react with a variety of fungi (Pisa et al., 2017).

Further evidence on the fungal origin of these structures was obtained using two additional rabbit polyclonal antibodies raised against purified fungal components: the polysaccharide chitin, a specific component of the fungal cell wall (Walker et al., 1990, 1991), and purified fungal enolase (Pisa et al., 2016b). Both antibodies immunoreacted with yeast-like cells in the striatum sections from the four patients analyzed (Supplementary Figure 2). As before, some of these rounded fungal cells were in close proximity to the cell nucleus. Of note, the chitin and enolase anti-fungal antibodies do not cross-react with human cells. Overall, the use of this panel of anti-fungal antibodies highlights the presence of fungal cells with different sizes and morphologies, perhaps reflecting a variety of fungal species in brain tissue.

**FIGURE 2:**
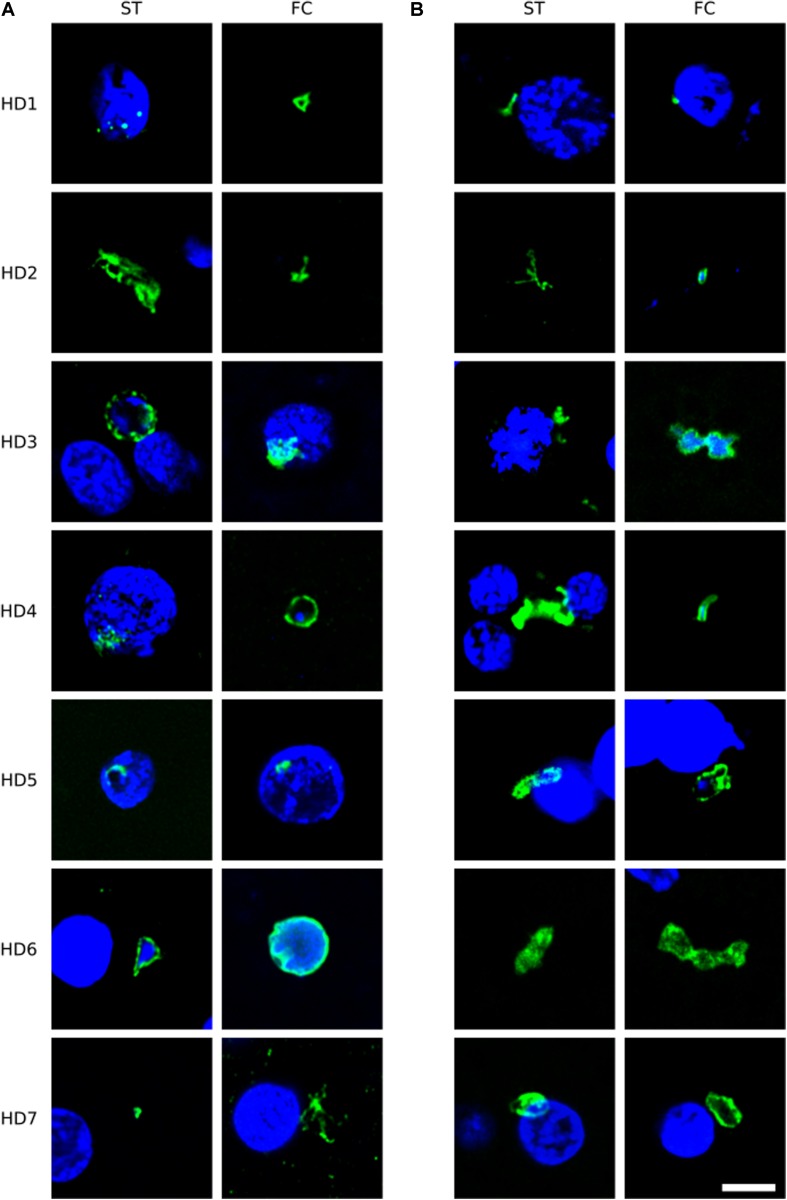
Immunohistochemistry of brain sections from seven Huntington’s disease patients using anti-*C. albicans* and anti-*S. racemosum* antibodies. Two CNS regions (striatum, ST and frontal cortex, FC) from seven HD patients (HD1–HD7) were processed for immunohistochemistry analysis. Paraffin sections were immunostained with rabbit polyclonal antibodies against *C. albicans* **(A)** or *S. racemosum* **(B)** (green). Nuclei were stained with DAPI (blue). Scale bar: 5 μm.

To broaden this analysis, brain sections from the FC and striatum from seven patients (HD1–HD7; grades I–IV) were examined using the anti-*C. albicans* antibody or a rabbit polyclonal antibody against *S. racemosum* (Figure 2). Consistent with the previous findings, several yeast-like cells were detected (green) and, in some instances, they were also positive for DAPI, indicating the presence of the fungal nucleus (Figure 2, panels A: HD3 ST, HD4 FC, HD6 ST, HD6 FC; panels B: HD2 FC, HD3 FC, HD4 FC, and HD5 FC). The size of some of these yeast-like cells was 4–7 μm, and showed an also showed a morphology different from that observed with the anti-chitin antibody. The different sizes of the fungal cells detected with each antibody may indicate the presence of a variety of species, which are preferentially recognized by each antibody. Figure 2 (panel B: HD5 FC) illustrates a sporulating yeast cell in which the nucleus is stained with DAPI (blue). Mycelial material was also evident, further demonstrating the presence of fungal infection (Figure 2, panels A: HD2 ST and HD7 FC; and panels B: HD2 ST and HD4 ST). These fungal structures were found both in FC and striatum sections. Altogether, these findings indicate that hyphal and yeast-like cells can be identified using a variety of anti-fungal antibodies in brain sections from the seven HD patients analyzed.

**FIGURE 3:**
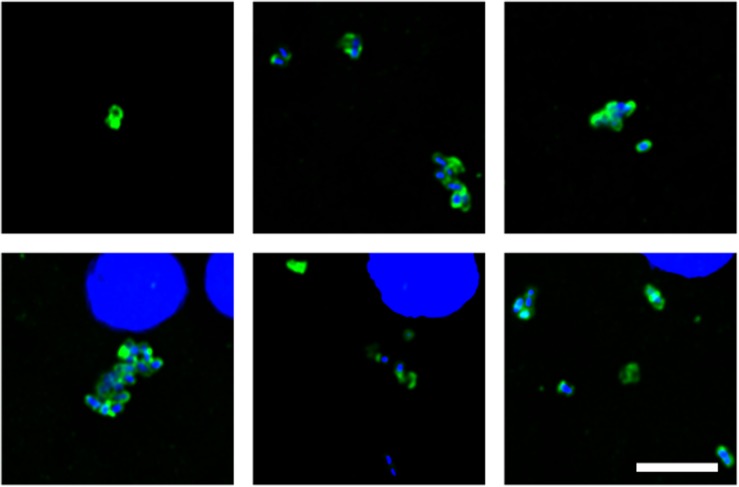
Fungal structures in the frontal cortex region from one Huntington’s disease patient (HD5) analyzed by immunohistochemistry. Immunohistochemistry analysis was carried out using polyclonal antibody against *C. albicans* (green). Nuclei were stained with DAPI (blue). Scale bar: 5 μm.

Of note, our attention was drawn to a group of elongated cells of about 1 μm in FC sections of patient HD5 (Figure 3). These yeast-like cells were immunopositive for *C. albicans*, supporting the idea that they are fungal in origin. Interestingly, this type of cell grouping was not observed in the other HD patients studied in this work, and it suggests that each HD patient might be colonized by different fungal species (see below).

**FIGURE 4:**
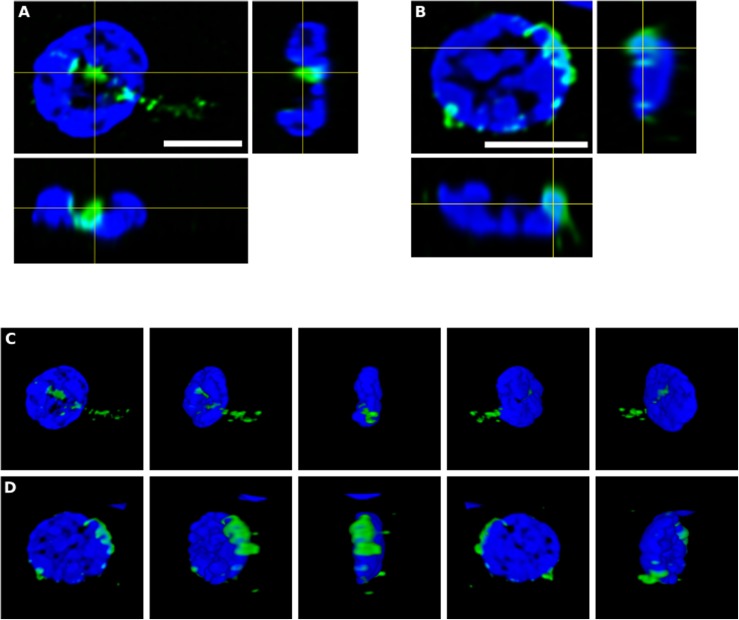
Orthogonal projection and 3D analysis of intracellular microbial structures. Immunohistochemistry was carried out as described in section “Materials and Methods.” Orthogonal projections **(A,B)** and different stacks from a 3D image **(C,D)** (see also Supplementary Movies 1, 2) from the striatum of HD6 patient. Panels **(A,C)** show the same cell that appear in panels **(B,D)**, respectively. Samples were immunostained with an anti-*C. albicans* antibody (green). Nuclei were stained with DAPI (blue). All scale bars: 5 μm.

To further assess the intracellular location of some of these fungal structures, orthogonal projections and 3D analyses were carried out (Figure 4). These intracellularly trapped fungi, known as endomycosomes, have also been observed in CNS tissue from patients with other neurodegenerative diseases (Pisa et al., 2015a; Alonso et al., 2017a). It was clearly evident that indeed some of these structures immunolabeled with anti-*C. albicans* antibodies (green) had projections to the nucleus, supporting the concept that the neural cells were infected when they were alive. Intracellularly located fungi positive for a range of anti-fungal antibodies are shown in Supplementary Movies 1–5, demonstrating their close connection with the nucleus.

### *Corpora amylacea* in Huntington’s Disease

**FIGURE 5:**
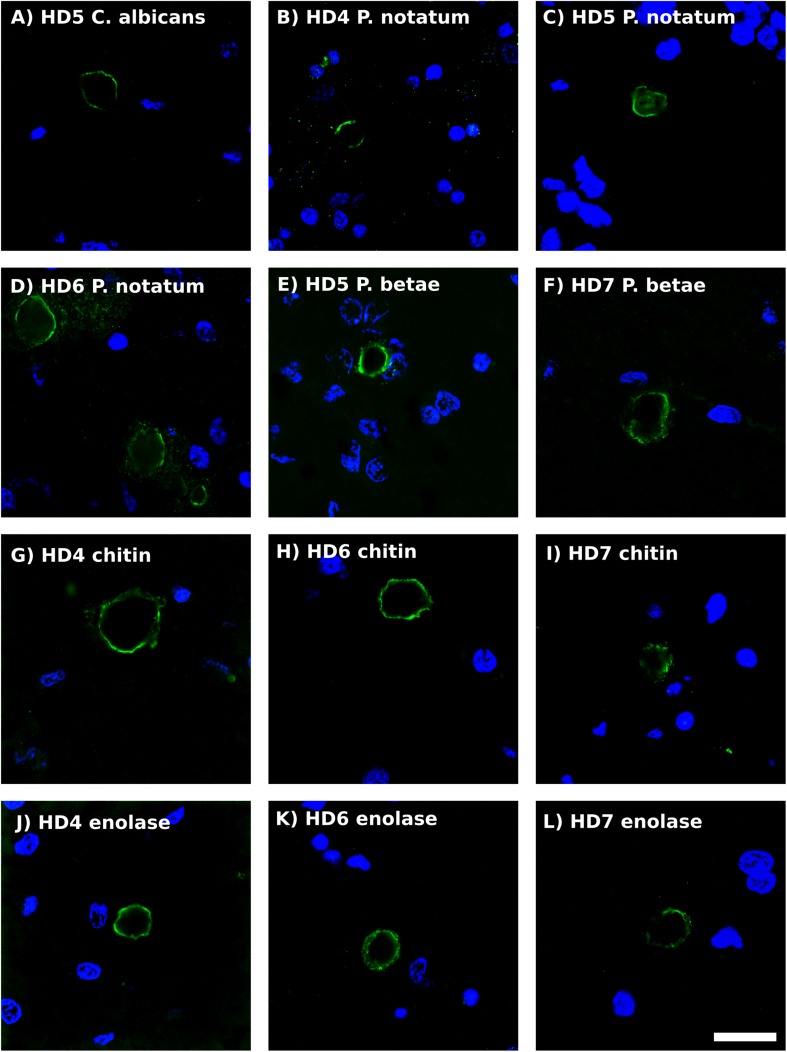
Analysis of *corpora amylacea* in Huntington’s disease brain sections by immunohistochemistry. Striatum sections from HD patients were incubated with rabbit polyclonal antibodies against *C. albicans* **(A)**, *P. notatum* **(B–D)**, *P. betae* **(E,F)**, chitin **(G–I)** and enolase **(J–L)** appear in green. **(B,G,K)** HD4; **(A,C,E)**: HD5; **(D,H,K)**: HD6; **(F,I,L)**: HD7. Nuclei were stained with DAPI (blue). Scale bar: 20 μm.

Previous studies from our laboratory have found fungal antigens in CA in patients with different neurodegenerative diseases (Pisa et al., 2016a, 2017; Alonso et al., 2018b). Using both immunohistochemistry and proteomic analyses, we have unequivocally identified fungal proteins in purified CA from patients with Alzheimer’s disease (Pisa et al., 2018). Although CA were described many years ago in the striatum from HD patients (Averback, 1981), to our knowledge very little is known about CA in HD. As shown in Figure 5, CA varying in size (10–40 μm) could be clearly identified in the striatum of the HD patients examined in this work, and these bodies immunoreacted with a variety of anti-fungal antibodies. In accord with our previous findings, the fungal antigens were mainly located in the envelope that surrounds a central portion of these spherical bodies. In some instances, immunoreactivity with the anti-*P. notatum* antibody was also found in the internal portion of CA (Figure 5, panel C). Immunoreactivity was also observed for antibodies against *C. albicans*, *P. notatum*, *P. betae*, chitin and enolase. These findings support the notion that fungal antigens are recruited, together with cellular debris, during the formation of CA. Of note, CA bodies were more easily identified in grades III–IV HD patients. We have previously proposed that CA may represent a response to microbial infections in the CNS of patients with neurodegenerative diseases (Pisa et al., 2016a, 2018). Since CA are built up during several months or even years, it seems plausible to believe that fungal infections are present during this time period long before death.

### Identification of Fungal Species in Huntington’s Disease Brains

**FIGURE 6:**
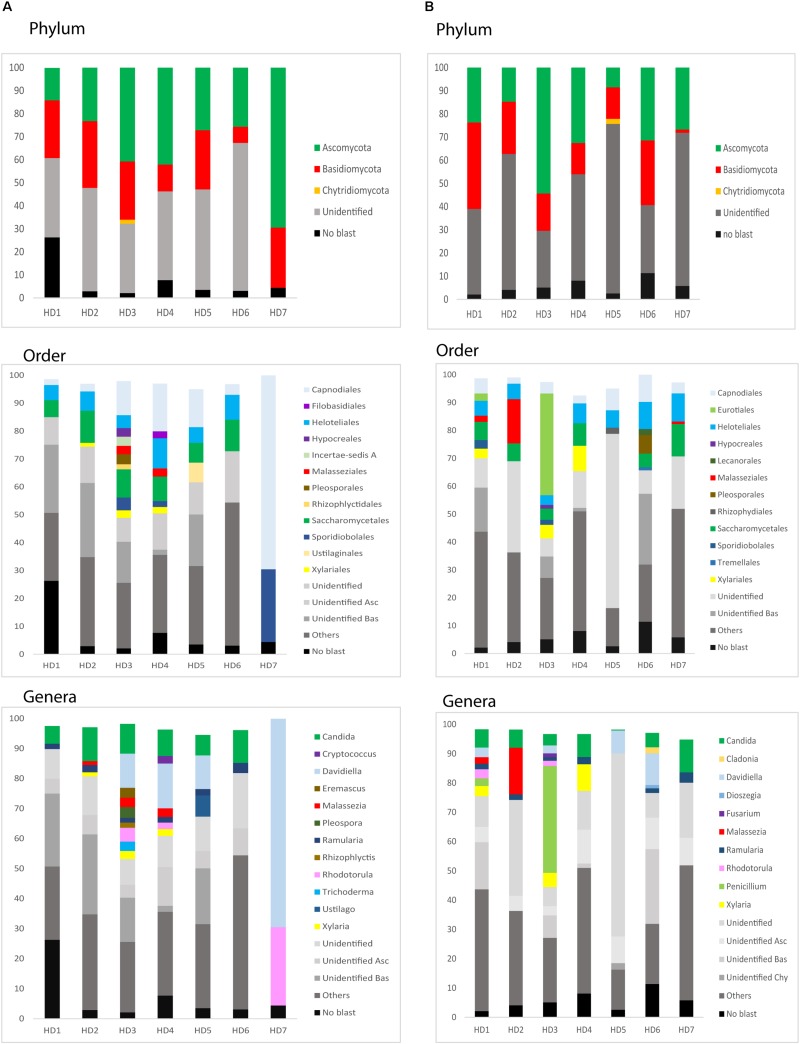
Identification of fungal phylum order and genus by next-generation sequencing. DNA was extracted from HD brain tissue for next-generation sequencing. Computational analyses of the sequences using QIIME classified the data into fungal phyla, order and genera. **(A)** Striatum tissue from seven HD brains. **(B)** Frontal cortex tissue from seven HD brains. Asc: *Ascomycota*, Bas: *Basidiomycota*, Chy: *Chytridiomycota.*

We next used NGS to precisely determine the fungal species present in HD brains. Extracted DNA from frozen tissue of both FC and striatum from patients HD1–HD7 was employed to amplify the fungal ITS1 located between the 18S and the 5.8S rRNA genes (as detailed in Materials and Methods). NGS was carried out using the Illumina platform, as indicated. The number of read sequences obtained for each sample varied between 1,18,143 and 2,13,301. Bioinformatic analyses of these sequences revealed a variety of fungal species, as detailed in Supplementary Table 3, which lists the fungal species above 1%. The fungal phyla order and genera in the two brain regions of the seven patients are shown in Figure 6 and the most prevalent fungal genera are listed in Table 1. These genera included *Candida, Davidiella, Malassezia, Rhodotorula*, and *Ramularia.* Of note, with the single exception of the genus *Ramularia*, these genera were also identified in brain tissue from other neurodegenerative diseases (Alonso et al., 2015, 2017a, 2018a; Pisa et al., 2018). It is possible that the tropism of *Ramularia* for HD patients makes this fungus more prevalent.

**TABLE 1:** Most relevant fungi detected in the frontal cortex and striatum from Huntington’s disease patients.

**ST**	**FC**
*Candida* (2.4–10.9%)	*Candida* (3.9–11.2%)
*Ramularia* (1.5–3.2%)	*Ramularia* (1.1–3.3%)
*Davidiella* (11.1–69.3%)	*Davidiella* (2.7–10.9%)
*Xylaria* (1.4–2.7%)	*Xylaria* (3.49%)
*Rhodotorula* (2.1–26.1%)	*Rhodotorula* (1.8–3.1%)
*Malassezia* (1.7–2.4%)	*Malassezia* (1.8–15.3%)

**FIGURE 7:**
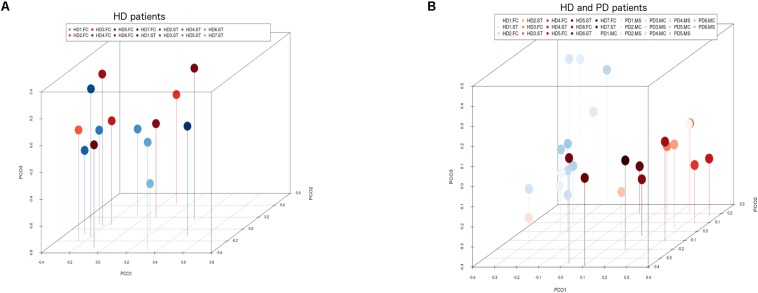
Principal component analysis of fungi identified by next-generation sequencing. 3D principal component analysis scatter plots of HD and Parkinson’s disease (PD) patients. Panel **(A)** shows the distribution between striatum (plots in blue) and frontal cortex (plots in red) regions of seven HD patients. Panel **(B)** shows the distribution between HD patients (plots in red) and PD patients (plots in blue). The UniFrac method was used to calculate this parameter. ST, striatum; FC, frontal cortex; MC, motor cortex; MS, mesencephalon.

Notably, PCA of the NGS data indicated that the distribution of these genera was similar between the FC and striatum regions (Figure 7, panel A). Interestingly, the distribution of fungal genera between patients with HD and those with Parkinson’s disease was significantly different (Figure 7, panel B). Overall, these results show that there are no major differences between the fungi present in the FC and striatum region in HD patients, whereas striking differences are evident between the fungi detected between HD and Parkinson’s disease. This finding is consistent with the notion that the mycoses in these patients with two different motor diseases differ.

### Analysis of Prokaryotic Structures in Huntington’s Disease Brains

**FIGURE 8:**
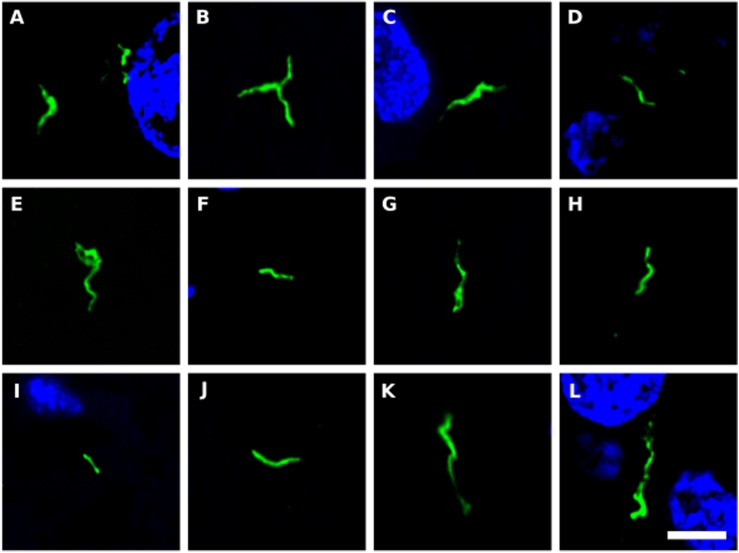
Prokaryotic structures in Huntington’s disease brain sections using an anti-*C. pneumoniae* antibody. Different sections from HD patients were incubated with a rabbit polyclonal antibody against *C. pneumoniae* (green). DAPI staining is shown in blue. **(A,C,E,F,H,J,K)** striatum region; **(B,D,G,I,L)** frontal cortex region; **(A,B)** HD1; **(C,D)** HD2; **(E)** HD3; **(F,G)** HD4; **(H,I)** HD5; **(J)** HD6; **(K,L)** HD7. Scale bar: 5 μm.

We next tested for the presence of bacteria in HD brain samples by immunohistochemistry using a battery of anti-bacterial antibodies. We failed to find any robust signal using the three commercial antibodies (see section “Materials and Methods”), with a rabbit polyclonal antibody and a mouse monoclonal antibody against *B. burgdorferi*. Likewise, no evident signal was found with a mouse monoclonal antibody against peptidoglycan. By contrast, a rabbit polyclonal antibody against *Clamydophyla pneumoniae* provided some findings of interest. In all seven HD patients examined, a curious curved morphological structure immunoreacted with this antibody (Figure 8). These curved filamentous structures of about 5–10 μm have not been detected by our group in brain samples from patients with other neurodegenerative diseases (Pisa et al., 2015a, 2017; Alonso et al., 2018b). The possibility that this morphology may correspond to a spirochete is tempered by the finding that it failed to immunoreact with an anti-*Borrelia* antibody.

### Identification of Bacteria in Huntington’s Disease Brain

In an initial attempt to detect prokaryotic DNA in brain tissue from HD patients, we used a PCR assay on DNA extracted from frozen samples, as indicated above for the detection of fungal DNA. First, PCR analyses were carried out using different primers that recognize prokaryotic DNA. One set of primers (universal) was directed to amplify a conserved region (V3–V4) of the 16S rRNA gene, whereas the other set amplified the IGS between the 16S and 23S rRNA genes. The universal primers rendered a DNA fragment of similar size independently of the bacterial species present in the sample, whereas the IGS primers rendered different sized fragments depending on the species detected. Interestingly, a DNA fragment of about 400 bp was present in all samples tested from the seven HD patients using the universal primers (Supplementary Figure 3, panel A). Sequencing analysis identified *Burkholderia cepacia* and uncultured *burkholderia* in most of these samples. Curiously, after sequencing the different fragments amplified by the IGS primers (Supplementary Figure 3, panel B), several bacterial species were identified, including *Cutibacterium acnes* (formerly known and *Propionibacterium acne*), *Escherichia coli* and *Streptococcus* spp. (Supplementary Table 4). These findings suggest that some species are preferentially recognized and amplified by the PCR assay depending on the primers and the PCR conditions. Finally, to test for the presence of spirochetes in the CNS samples from HD patients, we employed unique primers that detect this bacterial group. Control DNA extracted from *B. burgdorferi* rendered a DNA fragment of ∼350 bp using this assay (Supplementary Figure 3, panel C). However, no product was detected with the DNA samples from HD patients, pointing to the absence of spirochetes. Indeed, this finding is consistent with the results described above showing no immunoreaction with anti-*Borrelia* antibodies.

**FIGURE 9:**
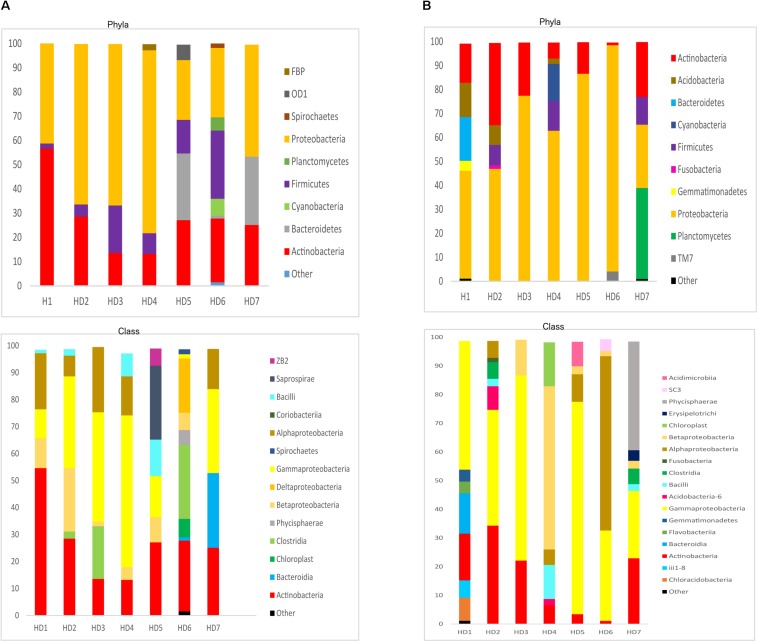
Distribution of bacterial phyla and classes in CNS samples from Huntington’s disease patients. Computational analyses of the sequences obtained using next-generation sequencing were carried out with QIIME. Bacterial phyla and classes obtained with this program are shown. Panel **(A)** shows bacterial phyla and classes detected in striatum of HD patients. Panel **(B)** shows bacterial phyla and classes detected from frontel cortex of PD patients.

We next used NGS to better assess the bacterial species in the HD brain samples. A great variety of bacterial species were identified both in striatum and FC regions from the seven patients (Supplementary Table 5). The most relevant bacteria are listed in Table 2. Notably, the genus *Burkholderia* was very prominent in some of the samples, in good agreement with the observations obtained by PCR analysis. Also, *Pseudomonas* and *Acinetobacter* were prominent in these patients. Indeed, the bacterial genera found in HD differed from the most prominent species detected in patients with Alzheimer’s disease or amyotrophic lateral sclerosis (Alonso et al., 2018a, 2019). The phyla and classes of the bacteria found in the striatum and FC are shown in Figure 9. Of note, proteobacteria and the classes gamma proteobacteria and *Actinobacteria*, were abundant in HD brain tissue.

**TABLE 2:** Most relevant bacteria detected in the frontal cortex and striatum from Huntington’s disease patients.

**ST**	**FC**
*Pseudomonas* (8.21–54.1%)	*Acinetobacter* (13.5–69.6%)
*Cutibacterium* (3.8–49%)	*Burkholderia* (49%)
*E. coli* (1.02–33%)	*Pseudomonas* (15.3%)
*Acinetobacter* (10.6–28.8%)	*Cutibacterium* (14.6%)
*Burkholderia* (7.24–17.5%)	*E. coli* (8.5–8.9%)

**FIGURE 10:**
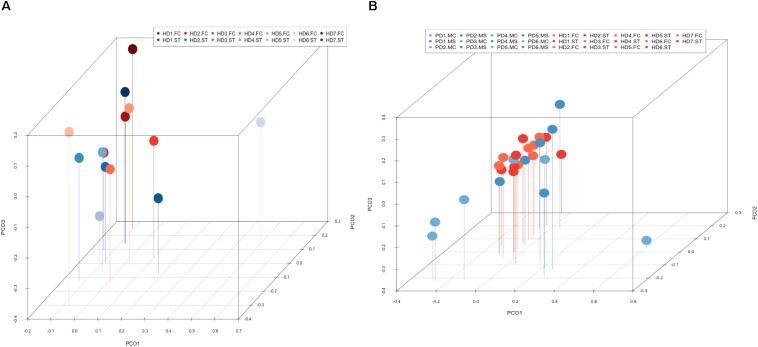
Principal component analysis of bacterial identified by next-generation sequencing. 3D principal component analysis scatter plots of HD and Parkinson’s disease patients. Panel **(A)** shows the distribution between striatum (plots in blue) and frontal cortex (plots in red) regions of seven HD patients. Panel **(B)** shows the distribution between HD patients (plots in red) and Parkinson’s disease patients (plots in blue). The UniFrac method was used to calculate this parameter. ST, striatum; FC, frontal cortex; MC, motor cortex; MS, mesencephalon.

PCA of striatum and FC results from the seven HD patients is illustrated in Figure 10, panel A. No discrimination of the prokaryotic species identified in the two CNS regions was detected by this analysis. Notably, when we compared the two CNS regions from HD with the mesencephalon and motor cortex regions from Parkinson’s disease patients, clustering of HD and mesencephalon from PD patients was evident. Curiously, a clear segregation of bacteria present in the motor cortex from PD brains was found. This finding supports the notion that the bacterial species of the striatum and FC from HD brains are similar to those detected in the mesencephalon from Parkinson’s disease patients. By contrast, the prokaryotic species of the motor cortex of Parkinson’s disease patients brains are different (Figure 10, panel B). This result supports the idea that bacteria present in brain microbiota in HD clearly differ from that observed in the motor cortex in Parkinson’s disease.

## Discussion

The main factor that determines HD is the CAG repeat expansion in the *HTT* gene, but the severity and progression of the disease can be shaped by other modulatory genes. In addition to *HTT* promoter alterations, genome-wide association studies have highlighted a number genes mainly involved in DNA repair as potential candidates to regulate HD (Lee et al., 2011; Becanovic et al., 2015; Moss et al., 2017). Also, epigenetic factors might regulate the progression and pathology of HD (Berson et al., 2018; Sallustio et al., 2019). In this regard, the present study is the first attempt to analyze in detail the brain microbiota in HD patients, which might constitute a risk factor that can contribute to the time of appearance of clinical symptoms. Microbial infections can play a part in the severity and progression of these symptoms. In this regard, it is noteworthy to find some fungal genera and some bacterial species that are more prevalent in HD as compared with other neurodegenerative diseases. This is the case for the genus *Ramularia*, which in concert with other fungi and bacteria can lead to alterations in neural cells. Also, our observation of a potential prokaryotic structure with a curved and filamentous morphology is typical of HD brains. Several bacterial genera such as *Pseudomonas*, *Acinetobacter*, and *Burkholderia* are more prevalent in HD than in Alzheimer’s disease or amyotrophic lateral sclerosis (Alonso et al., 2018b, 2019). Therefore, the specific microbiota of HD might participate in the neuropathology of the disease and might progressively colonize different areas of the HD brain that are prone to infection by some particular microbial species. This may be due to the fact that immune cells, microglia and other neural cells that synthesize the Huntingtin, facilitate this colonization. In addition, the tropism of these microorganisms for some brain regions could represent an important factor to explain the pathological findings observed in HD. The continuous production of extracellular enzymes (proteases, lipases, etc.) and also the synthesis of toxic compounds by some microbes could play an important part in the pathophysiology of HD. In this regard, mycotoxins, such as aflatoxins, ochratoxin A and trichothecenes can provoke a variety of cellular injuries, including the production of reactive oxygen species and mitochondria dysfunction (Yoon et al., 2009; Liu and Wang, 2016; Wang et al., 2017; Wu et al., 2017). In HD and other neurodegenerative diseases, mitochondria dysfunction and reactive oxygen species are usually detected in the CNS (Franco-Iborra et al., 2018; Elfawy and Das, 2019). It is therefore feasible that cellular alterations observed in patients with these diseases could be due to the continuous production of toxic molecules by microbes that progressively colonize the CNS. Consistent with this proposal should be the induction of general brain atrophy, selective tissue destruction and neural death observed in HD.

There are several arguments against the concept that post-mortem microbial colonization has occurred in HD patients: (1) the intracellular and even intranuclear location of some fungal structures indicates that infection took place in living cells. It is well established that fungi only enter metabolically active cells (Pacheco et al., 2007; Casadevall, 2008; Seider et al., 2014; Munoz-Duarte et al., 2016; Nelson and Nelson, 2018). (2) The presence of fungal antigens in CA is also consistent with the idea that these bodies entrapped fungal proteins during their formation over several months or years. (3) PCA demonstrates that the brain fungal mycobiome in HD is different from that in Parkinson’s disease. (4) Our previous studies using brain sections from control subjects did not show the variety of fungal structures observed in HD (Alonso et al., 2018b). Also, the putative bacterium with a curved and filamentous morphology described here has not been detected in brain sections from control subjects or in patients with other neurodegenerative diseases.

A salient clinical symptom in HD is neuroinflammation (Crotti and Glass, 2015; Rocha et al., 2016). Indeed, microglia and complement activation, as well as elevated levels of some interleukins, has been detected in HD brains (Singhrao et al., 1999; Bjorkqvist et al., 2008; Crotti et al., 2014). Moreover, these increased levels of interleukins and tumor necrosis factor are also found in the plasma of HD patients, even years before the disease is diagnosed, indicating systemic inflammation (Crotti and Glass, 2015; Politis et al., 2015). Increased circulating levels of other cytokines and pro-inflammatory molecules, such as C-reactive protein, are also detected in HD (Wang et al., 2014; Rocha et al., 2016). It is possible that the synthesis of mutated Huntingtin or the production of necrotic cell debris together with the aberrant protein can activate microglia and stimulate immune cells. Alternatively, based on our present findings it might be possible that neuroinflammation, microglia and complement activation are provoked by microbial infection. Likewise, the existence of infection would lead to a general stimulation of the immune system and the consequent elevated levels of cytokines in the plasma of these patients.

Another important characteristic in HD is the synthesis of amyloid (Mann and Jones, 1990; Chiti and Dobson, 2006; Porta et al., 2015; Shao et al., 2017) and it is noteworthy that amyloid peptide forms part of the innate immune system (Soscia et al., 2010; Kumar et al., 2016). Thus, it is possible that the synthesis of amyloid is triggered by microbial infection in HD. Notably, chitinase is also a biomarker for HD and appears in high levels in the CSF of patients (Vinther-Jensen et al., 2016). Chitinase, which is an enzyme synthesized by astrocytes and macrophages interacts with the polysaccharide chitin, which is a component of the fungal cell wall (Lee et al., 2011; Bonneh-Barkay et al., 2012; Gow et al., 2017).

Several scenarios can then be envisaged to help understand the pathology of HD. Since the most important feature of HD is the presence of CAG repeats in the *HTT* gene, it is possible that the accumulation of aberrant Huntingtin synthesized along several years could provoke cellular disfunction, and finally cell death. Considering the present findings, another possibility could be that dysfunction of the immune system and modifications in some tissues by mutated Huntingtin creates an environment conducive for colonization by some microbial species. In this respect, the initial infection by some microorganisms could also facilitate the entry of other opportunistic fungi and/or bacteria. In the second possibility, two alternatives can be considered. One is that brain colonization by microorganisms has no neuropathological effects. However, a more plausible alternative is that these polymicrobial infections can alter the natural physiology of neural cells. Moreover, our current observations provide an explanation for disease in those HD patients that do not harbor the CAG expansion repeat. In these patients without the expansion repeat, the clinical symptoms could be provoked by polymicrobial infections in some brain regions. As indicated above, the expression of mutated *HTT* can increase the probability for this microbial colonization, which could also take place in some instances even when normal Huntingtin, is synthesized. To distinguish between the different possibilities, clinical trials with safe anti-fungal and anti-bacterial compounds can aid to elucidate the role played by these infections in HD pathology.

## Data Availability Statement

Sequences have been submitted to the European Genome-phenome Archive with the accession number EGAS00001003678 and the European Nucleotide Archive with the accession number ERZ964645.

## Ethics Statement

The studies involving human participants were reviewed and approved by the Comité de Ética de la Investigación Universidad Autónoma de Madrid. The patients/participants provided their written informed consent to participate in this study.

## Author Contributions

DP performed the immunohistochemistry analyses. RA carried out the PCR and NGS analysis. LC designed the study and wrote the manuscript. All authors discussed the results and commented on the manuscript.

## Conflict of Interest

The authors declare that the research was conducted in the absence of any commercial or financial relationships that could be construed as a potential conflict of interest.

## Footnotes

1 http://www.bioinformatics.babraham.ac.uk/projects/fastqc/

2 http://qiime.org/
