# Supplementary material for: Brain Microbiota in Huntington’s Disease Patients
Source: Front Microbiol. 2019 Nov 12;10:2622. doi: 10.3389/fmicb.2019.02622 (PMC6861841; doi:10.3389/fmicb.2019.02622)
Supplement: Supplementary file 1 [file Data_Sheet_1.PDF]

BRAIN MICROBIOTA IN HUNTINGTON'S DISEASE PATIENTS

Ruth Alonso, Diana Pisa, and Luis Carrasco*

Centro de Biología Molecular "Severo Ochoa" (CSIC-UAM). c/Nicolás Cabrera, 1.

Universidad Autónoma de Madrid. Cantoblanco. 28049 Madrid. Spain.

+RA and DP contributed equally to this work.

*Corresponding author

Email address: lcarrasco@cbm.csic.es. Telephone number: +34 91 497 84 50

Running title: Huntington's disease and microbial infection

Key words: Huntington's disease; neurodegenerative diseases; polymicrobial infections; endomycosomes; fungal infection; next generation sequencing

Supplementary Figure 1. PCR analysis of the CAG expansion repeat and the hexanucleotide repeat of C9Orf72 in HD patients. PCR analysis was carried out as described in Materials and Methods. Panel A) PCR of DNA extracted from frozen tissue of the ST region from seven HD patients amplifying the CAG expansion repeat. Panel B) PCR of ST region from seven patients amplifying the hexanucleotide expansion repeat in the C9Orf72 gene. MW markers are shown on the left of the agarose gel. C - : PCR without DNA.

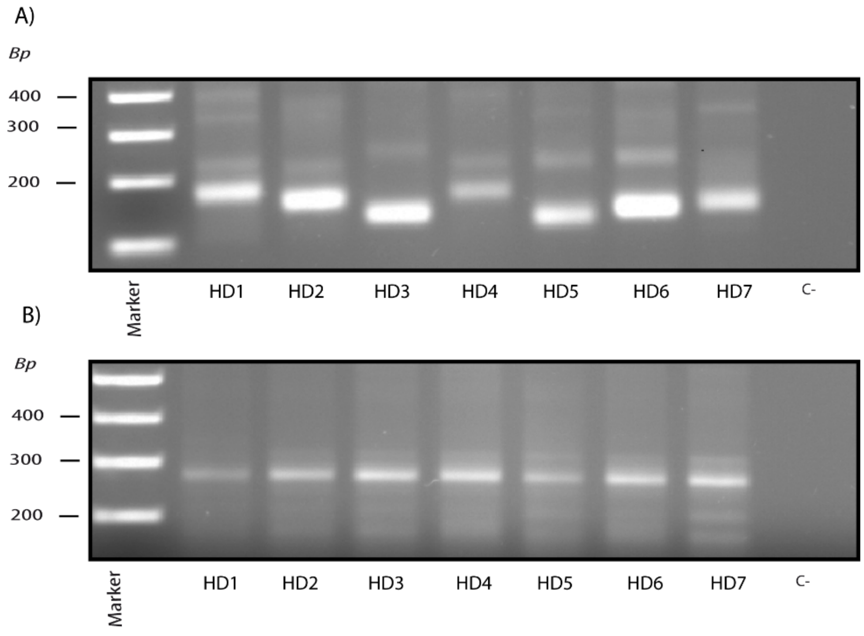

Supplementary Figure 2. Immunohistochemistry of striatum sections from HD patients using anti-chitin and anti-enolase antibodies. Striatum sections from four HD patients (HD4–HD7) were incubated using anti-chitin or enolase antibodies, respectively (green). Nuclei were stained with DAPI (blue). Scale bar: 5 µm.

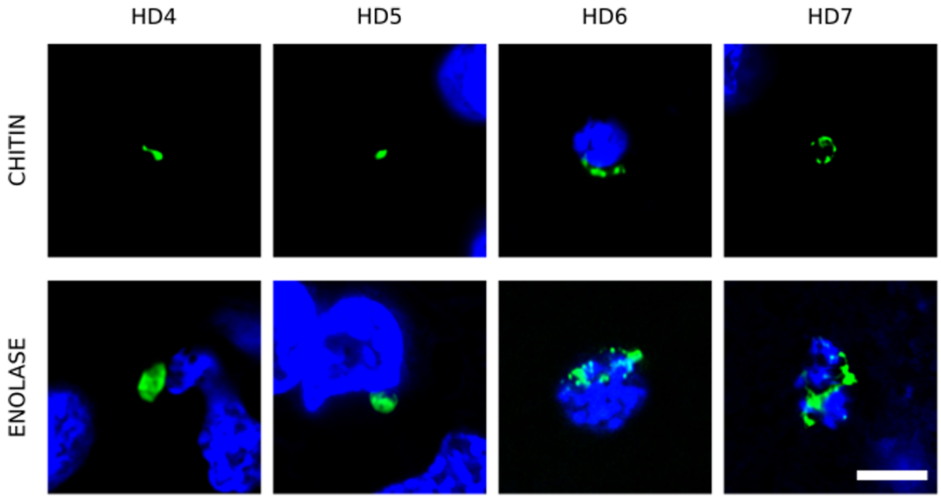

Supplementary Figure 3. PCR analysis of bacteria in DNA extracted from CNS regions of HD patients. PCR analysis was carried out as described in Materials and Methods. Panel A) Nested PCR analysis of ST and FC regions from seven patients amplifying the bacterial 16S rRNA gene using primers 27 (F) and 1492 (R) for the first round PCR and V3 and V4 for the second PCR. Panel B) Nested PCR analysis of two regions (ST and FC) from seven HD patients amplifying the IGS region. The primers 1406 (F) – 559 (R) and 1492 (F) – 242 (R) were used in the first and second round PCR, respectively. Panel C) PCR analysis to amplify the *flagellin* gene of *Borrelia burgdorferi* from seven HD patients. As positive control, DNA extracted from *B. burgdorferi* was used. MW markers are shown on the left of the agarose gel. Control - : PCR without DNA; CE, control of DNA extraction without DNA; ST, striatum; FC, frontal cortex.

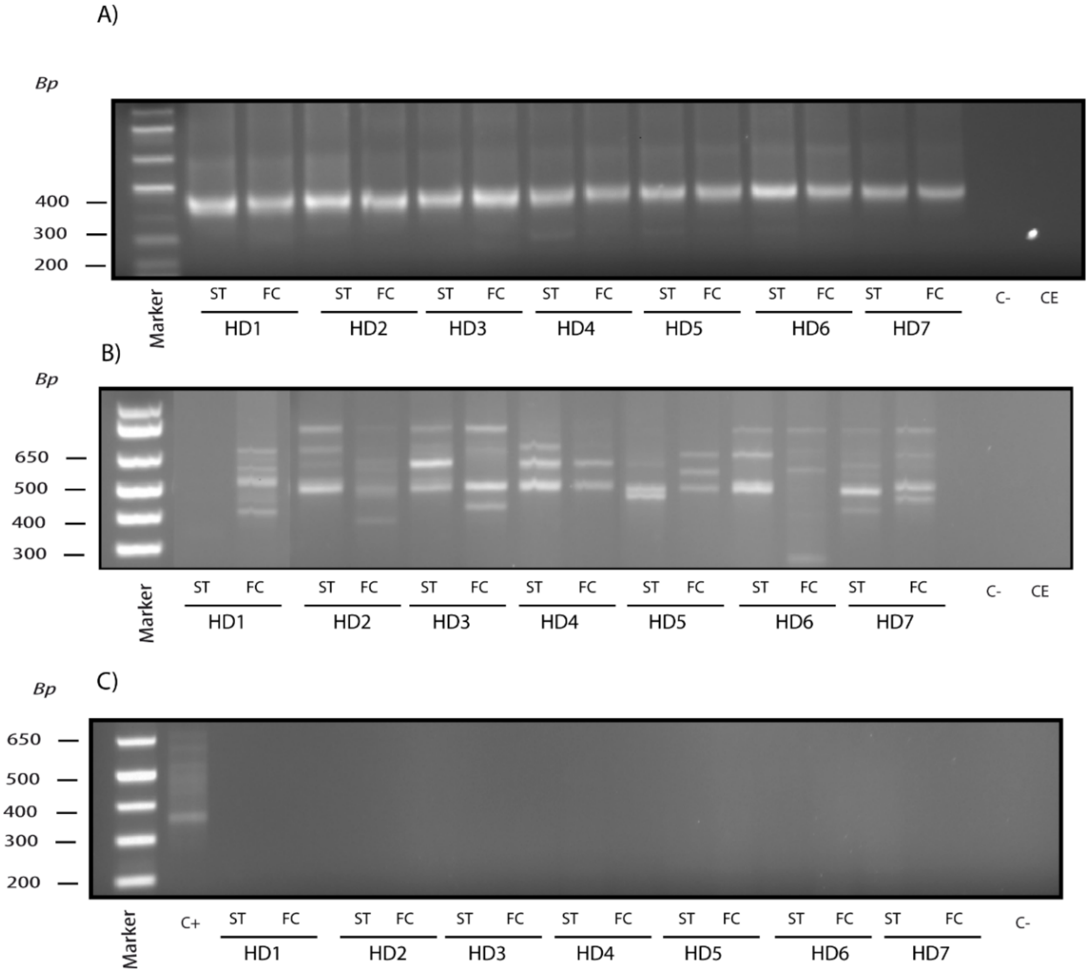

Supplementary Movie 1. Video images of intracellular fungi.

3D movie of Figure 4, panel C. HD6 ST sample was incubated with andi-*C. albicans* antibody shown in green, DAPI appears in blue.

Supplementary Movie 2. Video images of intracellular fungi.

3D movie of Figure 4, panel D. HD1 ST sample was incubated with anti-*C. albicans* antibody shown in green, DAPI appears in blue.

Supplementary Movie 3. Video images of intracellular fungi.

3D movie of HD3 ST sample was incubated with anti-*C. albicans* antibody shown in green, DAPI appears in blue.

Supplementary Movie 4. Video images of intracellular fungi.

3D movie of HD6 ST sample was incubated with anti-enolase antibody shown in green, DAPI appears in blue.

Supplementary Movie 5. Video images of intracellular fungi.

3D movie of HD5 ST sample was incubated with *P.betae* antibody shown in green, DAPI appears in blue.
