# Supplementary material for: Brain Microbiota in Huntington’s Disease Patients
Source: Front Microbiol. 2019 Nov 12;10:2622. doi: 10.3389/fmicb.2019.02622 (PMC6861841; doi:10.3389/fmicb.2019.02622)
Supplement: Supplementary file 2 [file Data_Sheet_2.pdf]

BRAIN MICROBIOTA IN HUNTINGTON'S DISEASE PATIENTS

Ruth Alonso, Diana Pisa, and Luis Carrasco*

Centro de Biología Molecular "Severo Ochoa" (CSIC-UAM). c/Nicolás Cabrera, 1. Universidad Autónoma de Madrid. Cantoblanco. 28049 Madrid. Spain.

+RA and DP contributed equally to this work.

*Corresponding author

Email address: lcarrasco@cbm.csic.es. Telephone number: +34 91 497 84 50

Running title: Huntington's disease and microbial infection

Key words: Huntington's disease; neurodegenerative diseases; polymicrobial infections; endomycosomes; fungal infection; next generation sequencing

Supplementary Table 1. Age and gender of HD patients.

PATIENT	GENDER	AGE	G. VONSATTEL
HD1	M	34	0-I
HD2	F	53	II
HD3	M	43	II
HD4	M	55	III
HD5	M	68	III
HD6	F	56	IV
HD7	M	64	IV

Supplementary table 2. Analysis of the CAG expansion repeat and the hexanucleotide repeat of C9orf72 in HD patients.

Patient	Normal alelle	Mutated allele	C9orf72
HD1	23	35	3
HD2	21	23	3
HD3	13	35	3
HD4	25	39	3
HD5	7	38	3
HD6	13	40	3
HD7	14	17+7	3

Sequence Normal Alelle (HD1)
TCATTAG**CAGCAGCAGCAGCAGCAGCAGCAGCAGCAGCAGCAGCAGCAGCAGCAGCAGCAGCAGCAGCAGCAGCAG**CAACCGCCGCCGCCGCCGCCGCCGCCGCCTCCTCCTCTTCTTCCGCCGACGAGATG
TTTATGCAGCTATTAGATCTAATGCTGGATCCTGTGACTCTAACATTGGCCGTGAAATCGATAAGGGAGG

Sequence Mutated allele:
GRAARRMR**CAGCAGCAGCAGCAGCAGCAGCAGCAGCAGCAGCAGCAGCAGCAGCAGCAG**CCGCCACCGCCGCCGCCGCCGCCGCCTCATCAGCTTCAGCAGACGAAG**CAGCAGCAGCAGCAGCAGCAG**CCG
CCGCCGCCGCCGCCGCCGCCGCCGCCGCCTCCGCAGCCGCAGCCGACGACGAAGTAACCTCACTCACATTAGTTGCAGCAGCGCTCACTGCTGATCTTTCCAGACAGCCAAACATGTACAAGCCAGCTGCATCAAAT
GAATAAGGCCAACGAACCGGAAGAGAAGAGGTTTGCTTATAACAGCAACACTTCCAACTTCCTCGCTGGAGTGACGACGCTACCCACCAAAGTTCGACTGCCGCGACCAGAAACAGCACGCTCGAAAGCAGGCTGGACG
GGCAATCACCCACAAACACAACGCGGAGGGAA

Supplementary Table 3. Fungal species detected by NGS in ST and FC from seven HD patients.

HD1ST		HD1FC		HD2 ST		HD2 FC		HD3 ST		HD3 FC	
Number of reads:118143		Number of reads:276036		Number of reads:179862		Number of reads:195990		Number of reads:186643		Number of reads:213301	
Sequence joined:88,1%		Sequence joined:96,7%		Sequence joined:95,4%		Sequence joined:95,5%		Sequence joined:98,6%		Sequence joined:97,9%	
unculturedBasidiomycota	24,34	unculturedBasidiomycota	15,82	uncultured Basidiomycota	26,4	uncultured zygomycete	22,3	uncultured Basidiomycota	14,6	Penicillium_aurantiogriseum	36,4
candida deformans	5,9	candida deformans	6,2	candida deformans	7,7	Malassezia_sp_HM_2008	15,3	Davidiella_tassiana	11,4	unculturedBasidiomycota	7,73
Uncultured Helotiales	4,8	Uncultured Helotiales	5,1	Uncultured Helotiales	6,3	candida deformans	6,2	candida deformans	5,1	Xylaria_curta	4,83
Uncultured fungus clone S24T_41	3,9	Uncultured fungus clone S24T_41	4,2	Uncultured fungus clone S24T_41	5,1	Uncultured Helotiales	5,1	Candida_sp,	4,82	candida deformans	3,9
Uncultured fungus clone CMH602	3,2	Xylaria_curta	3,489	Uncultured fungus clone CMH602	4,1	Uncultured fungus clone S24T_41	4,1	Uncultured Helotiales	4,2	Uncultured Helotiales	3,2
Ramularia glennii	1,7	Uncultured fungus clone CMH602	3,3	Candida_sp,	3,57	Uncultured fungus clone CMH602	3,3	Rhodotorula_mucilaginosa	4,1	Davidiella_tassiana	2,69
		Davidiella_tassiana	3,274	Ramularia glennii	2,3	Ramularia glennii	1,8	Pleospora_herbarum	3,57	Uncultured fungus clone S24T_41	2,6
		Rhodotorula_mucilaginosa	3,071	Xylaria_curta	1,4			Uncultured fungus clone S24T_41	3,4	Uncultured fungus clone CMH602	2,1
		Penicillium_aurantiogriseum	2,579					Eremascus_albus	3,33	unculturedsoil_fungus	1,83
		Ramularia glennii	1,8					Trichoderma_sp_CHTAM10	3,08	Rhodotorula_mucilaginosa	1,8
		Malassezia_sp_HM_2008	1,717					Xylaria_curta	2,75	Fusarium_oxysporum	1,32
								Uncultured fungus clone CMH602	2,7	Ramularia glennii	1,1
								Malassezia_sp_HM_2008	2,67		
								Rhizophlyctis_rosea	1,76		
								Ramularia glennii	1,5		
HD4 ST		HD4 FC		HD5 ST		HD5 FC		HD6 ST		HD6 FC	
Number of reads:194196		Number of reads:243144		Number of reads:208378		Number of reads:206556		Number of reads:156245		Number of reads:184219	
Sequence joined:98,3%		Sequence joined:98,4%		Sequence joined:98,4%		Sequence joined:97,9%		Sequence joined:97,3%		Sequence joined:98,3%	
Davidiella_tassiana	14,97	Xylaria_cubensis	9,04	unculturedBasidiomycota	18,5	unculturedendophytic_fungus	42	candida deformans	10,9	unculturedBasidiomycota	25,4

candida deformans	6,2	candida deformans	7,9	Davidiella_tassiana	11,2	Davidiella_tassiana	7,75	Uncultured Helotiales	8,9	Davidiella_tassiana	10,9
unidentified_sp,	5,236	Uncultured Helotiales	6,4	Ustilago_hordei	7,15	Rhizophydiales_sp_PL163L	2,22	Uncultured fungus clone S24T_41	7,3	Tubeufiaceae_sp_MFLUCC10_0115	6,65
Uncultured Helotiales	5,1	Uncultured fungus clone S24T_41	5,2	candida deformans	6,8			Uncultured fungus clone CMH602	5,8	candida deformans	4,9
Uncultured fungus clone S24T_41	4,1	ascomycete_sp_CH_Aa3L	4,959	Uncultured Helotiales	5,6			Ramularia glennii	3,2	Uncultured Helotiales	4,1
Uncultured fungus clone CMH602	3,3	Uncultured fungus clone CMH602	4,2	Uncultured fungus clone S24T_41	4,5					Uncultured fungus clone S24T_41	3,3
ascomycete_sp_IBWF79B_90A	2,552	Ramularia glennii	2,3	Uncultured fungus clone CMH602	3,6					Uncultured fungus clone CMH602	2,7
Cryptococcus_magnus	2,463	unculturedBasidiomycota	1,261	unculturedsoil_fungus	2,81					Cladonia_fimbriata	2,12
Candida_sp,	2,428			Ramularia glennii	1,9					Ramularia glennii	1,5
Malassezia_sp_HM_2008	2,375									Dioszegia_crocea	1,11
unculturedsoil_fungus	2,32										
Xylaria_curta	2,304										
Rhodotorula_mucilaginosa	2,104										
unculturedBasidiomycota	1,877										
Ramularia glennii	1,8										

HD7 ST		HD7 FC	
Number of reads:194617<br>Sequence joined:97,7%		Number of reads:201712<br>Sequence joined:97,5%	
Davidiella_tassiana	69,38	candida deformans	11,2
Rhodotorula_mucilaginosa	26,13	Uncultured Helotiales	9,2
		Uncultured fungus clone S24T_41	7,5
		Uncultured fungus clone CMH602	5,9
		Ramularia glennii	3,3

Supplementary table 4. Bacterial species IGS.

Species	Patients
Escherichia coli	HD3(ST);HD4 (FC)
Fusobacterium nucleatum	HD2 (FC)
Cutibacterium acnes	HD2(ST,FC); HD4(FC); HD5(FC); HD6(ST); HD7(ST)
Sthapylococcus aureus	HD4(ST)
Streptococcus pneumoniae	HD7(FC)
Streptococcus sp	HD1(ST)
Uncultured bacterium	HD2(ST)

Supplementary table 5. Bacterial species identified by NGS in FC and ST from HD patients.

HD1 ST		HD1 FC		HD2 ST		HD2 FC		HD3 ST		HD3 FC	
Cutibacterium acnes	43,59	*Acinetobacter schindleri*	12,9	*Escherichia coli*	33	*Acinetobacter lwoffii*	13	*Escherichia coli*	24,7	*Acinetobacter schindleri*	34
Phyllobacterium	19,61	*Bacteroides ovatus*	12,6	*Burkholderia*	17,5	*Arthrobacter*	12	*Corynebacterium*	11,6	*Cutibacterium acnes*	15
Burkholderia	8,716	*Arthrobacter*	11	*Rothia mucilaginosa*	12,2	*Corynebacterium*	12	*Sphingomonadales*	11,3	*Betaproteobacteria*	12
Pseudomonas viridiflava	4,517	*Acinetobacter lwoffii*	9,88	*Microbacterium*	8,34	*Pseudomonas pseudoalcaligenes*	6,8	*Blautia*	10,9	*Escherichia coli*	8,9
Corynebacterium	3,413	*Escherichia coli*	8,44	*Rhizobiales*	3,89	*Pseudomonas viridiflava*	6,8	*Anaerococcus*	7,95	*Moraxella*	8,4
Corynebacteriumkroppenstedtii	2,671	*RB41*	7,92	*Phyllobacterium*	2,67	*Acidobacteria*	6,2	*Acinetobacter lwoffii*	7,73	*Corynebacterium kroppenstedtii*	6,1
Cutibacterium	2,268	*iii1-8*	6,03	*Burkholderiaceae*	2,32	*Actinomycetales*	4,9	*Sphingobium sp*	4,24	*Enterobacteriaceae*	3,4
Burkholderiaceae	1,493	*Gemmatimonadetes*	4,09	*Veillonella dispar*	2,04	*Cellvibrio*	4,7	*Sphingobium xenophagum*	3,16		
Actinomycetales	1,146	*Gillisia*	3,86	*Ralstonia*	1,32	*Roseburia*	3,3	*Xanthomonadaceae*	2,55		
Pseudomonas	1,089	*Enterobacter cloacae*	3,33	*Mycobacterium*	1,29	*Pseudomonas*	2,6	*Alphaproteobacteria*	2,3		
Cutibacterium granulosum	1,082	*Rothia mucilaginosa*	2,61	*Scardovia*	1,26	*Corynebacterium*	2	*Acinetobacter schindleri*	1,56		
Escherichia coli	1,019	*Haemophilus parainfluenzae*	2,41	*Streptococcus*	1,19	*Acidobacteria-6*	1,9	*Sphingobium*	1,17		
		Enterobacter	1,63	*Salinispora*	1,15	*Rhodobacter*	1,8	*Acinetobacter*	1,15		
		Klebsiella	1,29	*Corynebacterium kroppenstedtii*	1,1	*Corynebacterium kroppenstedtii*	1,5	*Phenylobacterium*	1,13		
		Other	1,12			*Sphingomonas*	1,5	*Ralstonia*	1,08		
		Acinetobacter;Other	1,09			*Fusobacterium*	1,4				
		Enterobacteriaceae	1,06			*Micrococcaceae*	1,1				
						Gammaproteobacteria	1				
